# Diffusion tensor imaging for Alzheimer’s disease: A review of concepts and potential clinical applicability

**DOI:** 10.1590/S1980-57642009DN30400002

**Published:** 2009

**Authors:** Luciano de Gois Vasconcelos, Sonia Maria Dozzi Brucki, Andrea Parolin Jackowiski, Orlando Francisco Amodeo Bueno

**Affiliations:** 1 MD, Post-Graduate Student of the Psychobiology Department, UNIFESP, Brazil; 2 MD, PhD, Affiliated researcher of the Psychobiology Department, UNIFESP, Brazil; 3 PhD, Professor of the Psychiatry Department, UNIFESP, Brazil; 4 PhD, Professor of the Psychobiology Department, UNIFESP, Brazil.

**Keywords:** Alzheimer’s disease, magnetic resonance imaging, early diagnosis, review

## Abstract

In view of the urgent need to identify an early and specific biomarker for Alzheimer’s disease (AD), a PubMed database search was performed using the terms “Alzheimer disease” and “Diffusion Magnetic Resonance Imaging” to enable review of Diffusion tensor imaging (DTI) concepts and its potential clinical role in AD evaluation. Detailed analysis of selected abstracts showed that the main DTI measures, fractional anisotropy and apparent diffusion coefficient, indicators of fiber tract integrity, provide a direct assessment of WM fibers and may be used as a new biomarker for AD. These findings were found to correlate with cognitive assessments, rates of AD progression and were also able to differentiate among groups including mild cognitive impairment, AD, and other dementias. Despite several consistent DTI findings in AD patients, there is still a lack of knowledge and studies on the DTI field. DTI is not yet ready for clinical use, and requires extensive further research in order to achieve this goal.

## Abstract (pt)

Considerando a urgência em se determinar biomarcadores específicos e precoces da doença de Alzheimer (DA), realizou-se pesquisa bibliográfica no banco de dados Pubmed com os termos “Alzheimer disease” e “Diffusion Magnetic Resonance Imaging” objetivando-se revisar conceitos e utilidades clínicas da imagem do tensor de difusão (DTI) na avaliação da DA. A análise dos resumos selecionados mostrou que as principais medidas da DTI, anisotropia fracional e coeficiente de difusão aparente, indicadores de integridade dos feixes nervosos fornecem uma análise direta das fibras de substância branca e podem ser utilizadas como biomarcadores da DA. Estes achados correlacionaram-se com avaliações neuropsicológicas, taxa de progressão da DA e identificou grupos heterogêneos como declínio cognitivo leve, DA e outras demências. Apesar dos vários e consistentes achados da DTI na DA, ainda tem-se carência de conhecimento e pesquisas nesta área. DTI não está pronto para o uso clínico e muito trabalho deve ser realizado para obtenção deste objetivo.

Dementia has already been established as one of the major health challenges of this century due to the burden these pathologies impose on health care systems. Dementia is a public health problem in many parts of the world, including developed^1^ and developing countries such as Brazil.^2-4^ It is one of the most common diseases in the elderly and a major cause of mortality, disability, loss of quality of life, and a serious economical threat to our societies.^5^

Alzheimer’s disease (AD) is a leading cause of dementia among the elderly and currently no effective treatment exists that can significantly alter the course of the disease.^6^ Substantial effort is currently being focused towards improving the diagnosis and treatment of AD.^7^

Early intervention with disease-modifying therapies is likely to be more effective in cases with a lower burden of amyloid and hyperphosphorylated tau, and can attenuate the negative effects of secondary events caused by deposit of these toxic products in tissue.^8^ Identifying distinctive findings of early AD and of prodrome states might allow earlier and potentially more specific diagnosis, treatment and clinical delay of dementia manifestations.^9^

The current diagnostic process in early dementia stages is typically based on detecting subtle changes. Additional substantial diagnostic challenges are often faced by physicians, especially when the presence of memory and functional deficits are mild or ambiguous and the informant is unable to provide a clear history of deterioration from a pre-existing level of cognitive-functional status.^10^ Although patients are presenting earlier in the course of the disease, a high level of accuracy can only be achieved when patients are followed over many years and assessed by multiple tools.^11^

There are several obstacles to defining mild cognitive impairment (MCI) including a very wide range of annual rates of progression to AD (5-16%), low specificity and sensitivity, and selection of heterogeneous profiles of patients (30% of MCI patients exhibit non-AD pathology).^8^

Current diagnostic criteria lack specificity. When using standard references, DSM-IV-TR and NINCDS-ADRDA have an accuracy ranging from 65-96%, while the specificity of these diagnostic criteria amongst other dementias is only 23-88%.^8^

Further hampering the diagnostic process, morphological neuroimaging changes related to normal aging are also seen in AD patients, introducing overlap and thus limiting the expected diagnostic sensitivity and specificity.^12,13^

Given the frailties of pure clinical diagnostic criteria for both MCI and AD, some authors^8^ have suggested the use of biomarkers [genetic testing, molecular imaging, body-fluid biomarkers and other Magnetic Resonance Imaging (MRI) - based neuroimaging techniques] to improve diagnosis, recognizing that present criteria often fail to properly distinguish early AD from others differential diagnoses. These current clinical criteria also attenuate the clinical trials results by identifying heterogeneous populations.^11^

Assessment of the efficacy of these technologies in detecting preclinical and prodromal AD stages may prove the clinical value of these tools.^14^ Advanced MR techniques, such as diffusion tensor imaging (DTI), can detect early microstructural alterations in AD patients before gross anatomic alterations become visible,^15^ alterations which are generally not detected by conventional MRI.^16^

Because MR scanners are widely available, non-invasive, and reliable^6^ there is increasing interest in using DTI for diagnosing AD.^17^ DTI potentially meets all these qualities,^18^ as recommended by a previous consensus conference.^19^

The relevance of AD, clinical evaluation difficulties, weakness in current diagnostic criteria, the urgent need for early AD diagnosis and limited DTI usage in this clinical context, were the main reasons for reviewing the concepts of DTI and its clinical applicability to AD management.

## DTI concepts

Recent years have been marked by increased usage of advanced magnetic resonance techniques, which provide additional information beyond macroscopic morphology. By employing the scanner hardware in different ways, magnetic resonance spectroscopy, functional MRI, and DTI can provide information on brain metabolism, neural activity, and tissue microstructure, respectively.^13^

Conventional MRI traditionally played an exclusionary role, in ruling out secondary causes of cognitive decline. The technique was not provided any specific support to discriminate among different forms of dementia. Clinical research has explored the macroscopic measures of brain to identify possible biomarkers. However, these measures reveal only the final stage of dementia process, proving insufficient for the purposes of early diagnosis.

Diffusion MRI,^20^ better known as two distinct classes of application, Diffusion Weighted MRI (DWI) and DTI, has shown the ability to detect subtle changes in brain tissue. These methods indirectly identify microscopic aspects that provide measures reflecting the patterns in size, orientation and organization of tissue which are supposed precursors to the final stage of macroscopic tissue atrophy.^21^

DWI of the brain was first used during the early nineties and found to have immediate utility for the evaluation of suspected acute ischemic stroke. Since then, the technology of diffusion imaging has led to greatly improved image quality and enabled many new clinical applications. DTI and fiber tractography have advanced scientific understanding of many neurologic and psychiatric disorders, having been applied clinically for presurgical mapping of white matter pathways to avoid postoperative injury and proved useful for the diagnosis of intracranial pyogenic infections, demyelinating disease, masses, trauma, and vasogenic-versus-cytotoxic edema.^22^

In DWI, each image voxel has an image intensity that reflects a single best measurement of the rate of water diffusion at that location. DWI is most applicable when the tissue of interest is dominated by isotropic water movement and the diffusion rate appears to be the same when measured along any axis.^20^

DTI is valuable when a tissue has an internal fiber structure analogous to the anisotropy of some crystals, such as the white matter fiber tracts in the brain. Water tends to diffuse more rapidly in the direction of the internal structure and more slowly as it moves perpendicular to the passage of least resistance. The measured rate of diffusion differs depending on the direction from which is observed. Each voxel therefore has one or more associated pairs of parameters: a rate of diffusion and a preferred direction of diffusion.^20^

The properties of each voxel of a single DTI image is usually calculated by vector or tensor math from six or more different diffusion weighted acquisitions, each obtained with a different orientation of the diffusion sensitizing gradients. In some methods, hundreds of measurements are made to generate a single resulting calculated image data set. The higher information content of a DTI voxel makes it extremely sensitive to subtle pathology in the brain. The directional information can be exploited at a higher level of structure to select and follow neural tracts through the brain, a process called tractography.^20^ Detailed review of the physical aspects of DWI and imaging techniques characteristics are described in relevant articles.^22-25^

DTI provides derived quantitative measures which reveal cellular microstructure and fiber tract integrity. The main measures are fractional anisotropy (FA), apparent diffusion coefficient (ADC), and mean diffusivity (MD).^26^

Cerebral white matter bundles are arranged in a highly directional and packed manner. The diffusivity of water is hindered mostly by axonal membranes, and by myelin sheaths. The water diffusivity is therefore much higher along the direction of the bundle compared to other directions, a condition known as anisotropic diffusion. The difference between diffusivity along and across bundles increases with axonal density. Several measures of diffusion anisotropy exist, the most common being known as FA.^13^ FA describes fiber tract directionality and provides an index of the tendency for water diffusion to occur in a single direction within a voxel. FA is measured on a scale from 0 (random diffusion) to 1 (highly linear diffusion).^27^ The diffusion tends to flow parallel to the direction of fiber tracts and disruption of these tracks can reduce this tendency.^27^ Values therefore reflect the density of axons within bundles, with lower values corresponding to lower axonal density.^13^

ADC represents the interaction of the diffusing molecules with cellular structures and determines overall diffusivity. In the non-colinear direction or free diffusion, this constitutes a scalar measure of water diffusion^5^ and does not assess the directionality of molecular movement.^27^ However, it can be considered a crude measure of barrier density and therefore of cellularity, with denser tissues showing lower ADC values.^13^ MD provides the same information as expressed by the ADC.

Image processing is of vital importance in DTI with numerous programs available to users that provide a computerized approach for tensor calculation, color mapping, fiber tracking, ROI analysis and three-dimensional visualization.

Once the FA and MD maps are generated from these programs, post-processing can begin. At present, there are three documented approaches to DTI post-processing, namely, manual region-of-interest (ROI)-based, voxel-based analysis and tract-based spatial statistics (TBSS). These methods are in some ways similar to the methods used for volumetric evaluation in quantitative MRI studies.^26^

Some studies have employed ROI-based approaches in selected brain areas.^5^ In vivo fiber tracking can be performed by using the multivariate information from diffusion tensors. This allows reconstruction of the fiber tracts originating from selected white matter areas based on individual DTI scans. DTI-based tractography is conceptually similar to ROI analysis, but in the latter, ROIs are represented by fiber tracts that are automatically defined by tractography algorithms. This method is less operator-dependent compared to ROI analysis.^21^ More detailed information on DWI imaging acquisition techniques and post-process analysis are available in other relevant articles.^28^

## Objectives

To review the concepts of diffusion tensor imaging, and its potential clinical applicability in evaluating Alzheimer’s disease.

## Methods

Data were drawn from the PubMed database using the search terms ‘Alzheimer’s disease’ and ‘Diffusion Magnetic Resonance Imaging’. The following filters were applied: Entrez Date from 1999/05/30 to 2009/05/30; Publication Date from 1999/05/30 to 2009/05/30; only items with abstracts; Humans.

The DWI clinical applicability analysis was based on selected abstracts from the full search. The selection was based on the following criteria: articles in the English language and usage of any DWI technique in humans and involvement of at least one study group of AD patients. Some abstracts retrieved in our search, of studies on mild cognitive impairment patients only, were also selected.

Additional papers, besides the articles selected in the initial search, were also used mainly for the development of the introduction and discussion sections.

## Results

The search of the Pubmed database retrieved 110 abstracts. Of these, 56 abstracts were selected for in-depth analysis. A table describing the pertinent aspects of the analysis is available online (www.demneuropsy.com.br).

Forty articles (71%) were published from 2005 to 2009, and the median number of participants recruited for studies was 34. Of the 56 abstracts assessed, 2 studies involved longitudinal follow up of the participants (ranging from 15 to 29 months) and 54 had a cross-sectional design. Different methods of DTI data acquisition and post-processing were employed by the studies.

Abstracts selected compared DTI findings among AD, MCI or elderly healthy controls. Few papers compared AD with others diagnoses such as Parkinson’s disease (PD), Parkinson’s disease dementia (PDD), posterior cortical atrophy (PCA), vascular dementia (VD), and Lewy Body Disease (LBD).

The main DTI measures used were FA, ADC and MD. Others measures were occasionally cited such as radial diffusivity and axial diffusivity.

The most relevant encephalic regions assessed were temporal areas, parietal areas, frontal areas, posterior areas, and some integrative intracortical fiber tracts. Specific structures such as the corpus callosum, cingulate gyrus/cingulate fiber tracts, uncinate fasciculus, inferior longitudinal fasciculus/superior longitudinal fasciculus fiber pathways and hippocampus were studied. Other structures were cited as secondary including thalamus, basal ganglia, and internal capsule.

The main DTI measures (FA and ADC) of the above-mentioned encephalic structures showed compromised tissue integrity and marked changes in relevant fiber tracts, from very early AD stages. These findings were found to correlate with cognitive assessments, and also differentiated between groups such as normal ageing, MCI, AD, and others dementias.

## Discussion

Neuroimaging is a stimulating tool for investigation of the epidemiology, diagnostic efficacy, rate of progression, therapeutic effects, and offers considerable potential to explain the earliest functional changes in AD and other dementias^14,16^. Most studies of these emerging technologies are at the developmental stage, exploring the underlying disease process and defining differences across various subject groups.^14^

A number of biomarkers, imaging techniques and neuropsychological tests are under development to improve the assessment of cognitive decline and have produced promising results. However, insufficient evidence is available regarding their sensitivity, specificity, reproducibility, ease of use or whether these instruments have an impact on the final outcome of these patients.^11,29^

Neurochemical (T-tau, P-tau, Aβ42) and MRI-based markers (hippocampal and whole brain volumetry) are currently being assessed in multicenter controlled diagnostic studies (advanced phases) to determine the sensitivity and specificity of the markers and to perform an initial assessment of their positive and negative predictive values. Other biomarkers are in the early phases of assessment, particularly analysis of cortical thickness, deformation-based morphometry, voxel-based volumetry, DTI, spectroscopy, and fMRI. Advanced phases studies are underway to investigate whole brain volumetry as a secondary end point in several clinical trials. The validity of this marker however, is limited.^5,30^

The most recognized structural biomarker of AD is hippocampus volume, already implemented as a measure of therapeutic effect on disease modification in AD. Automated approaches are being developed as an endpoint of clinical trials given the interest in these methods expressed by the regulatory authorities. DTI is a developing area in which analysis techniques include optimal use of multivariate data. Consistent data on changes of the fiber tract in early AD are still incomplete, but results have been promising.^5^

DTI is unique in providing quantitative maps reflecting the density of axonal bundles which greatly improves the availability of connectivity data, while its noninvasive nature enables longitudinal studies to be performed. Spectroscopy, functional MRI, and DTI are inherently complementary in integrating morphological imaging and morphometric measurements. Macroscopic findings elucidated by morphological imaging can be integrated by DTI, whose main measures are potentially earlier indicators of degeneration than volume loss.^13^

Studies have shown that diffusivity generally tends to be higher in AD patients and intermediate in patients with MCI, characterized by greater deterioration especially in temporal structures. Strong correlation of diffusional measurements and neuropsychological scores has been found in cognitively impaired elderly. Positive correlation between cognitive performance and fractional anisotropy, and negative correlation between cognitive performance and mean diffusivity have also been described.^13^

Diffusion MRI studies have demonstrated that in addition to cortical changes, microscopic white matter changes occur in patients with AD, which are undetectable by conventional MRI. Knowledge of the pattern of WM microstructural changes in AD and its underlying mechanisms may contribute to earlier detection and intervention in groups at risk for AD.^31-33^

DTI analyses in AD or MCI have demonstrated brain structural disturbances predominately in regions commonly affected in early AD including the hippocampal area, temporal area, posterior cingulate, and corpus callosum. A significant correlation among overall or regional diffusivity, and anisotropy and global cognitive status has been repeatedly described.^34-37^

Other findings have highlighted correlation among damage to WM tracts in several corticocortical and corticosubcortical connection areas, and clinical and neuropsychological features of the different forms of degenerative dementias, from early phases of disease.^15,21^ Several studies have demonstrated an antero-posterior gradient in age-related reduction of density of axonal bundles, mainly in frontal regions and corpus callosum.^13^ Some results highlight that hippocampal microstructural changes could be a better predictor of risk of progression of MCI to AD than hippocampal atrophy. These changes may be apparent earlier than the macroscopic changes measured on hippocampal volumetry and were associated with poor cognitive functioning.^38,39^

Classification of white matter into white matter hyperintensities (WMH) and normal appearing white matter (NAWM) may be arbitrary and even inappropriate. DTI and histopathological measures suggest that a continuum may exist from neuropathologically normal to abnormal white matter. DTI and T1 measurements were shown to be more specific than conventional T2-weighted MRI, as these techniques revealed differences between WMH of demented and non-demented subjects, and reflected the severity of the underlying neuropathological changes.^40^

A number of methods of DTI imaging analysis have been proposed and applied.^21^ Despite efforts to standardize DTI data acquisition and analysis protocols,^41^ some problems are still evident such as data variability across centers,^42^ lack of uniform methods for acquisition and post-processing of DTI data, and unknown impact on the outcome of patients with AD.^29^

The greatest difficulty in comparing DTI studies is the lack of a commonly agreed standard for placement of regions of interest for statistical analysis, combined with inter-individual variability in the layout of fiber bundles.^13^

A minimum of technological resources and trained staff should be available when DTI imaging is considered. This method should be performed by multidisciplinary teams including neuroscientists, physicists, and engineers in order to provide clinically viable data. Such specialists are currently available in only a small proportion of MRI centers worldwide. Clinical and research applicability should be determined in the context of these limitations.^13^

Attention should also be paid to ensure that these sophisticated and limited tools yield their expected benefits and do not become a barrier to treatment, especially in developing countries where access to technology is limited and dementia is an emerging problem.^11^

Research areas for developing DTI usage and clinical application include: development of the analysis methodology of DTI-based tensor maps and assessment of multivariate nature of the data obtained; clinical application of these findings to AD research and possibly for studying connectivity and plastic organization of the human brain; role in the differential diagnoses of dementias, such as vascular dementia and AD; potential diagnostic benefits of combining different methods of neuroimaging; longitudinal follow up studies of DTI results during AD and MCI evolution.^5^

A practical approach is advised when considering the role of these techniques in the clinical practice. The burden that the elderly population is placing on health-care systems is such that techniques which do not significantly affect outcome are rejected. Therefore, treatment development that will ultimately be the driver determining the clinical impact and dispersion of DTI.^13^

## Conclusions

The clinical applicability of DWI techniques in AD, particularly DTI, remains limited. The few studies available are both recent and superficial.

Some consistent DTI findings have been found in AD patients, despite the limitations cited. For instance, FA and ADC, indicators of fiber tract integrity, may be used as AD biomarkers.

Further DTI studies with a longitudinal design are needed to investigate AD and other dementias.

The lack of uniform usage of standard methods of acquisition and post-processing of DTI images hampers the comparison of results among different research centers.

The timing is right to use the advanced technology available to improve the early, accurate diagnosis of dementia and to develop more effective interventions.

In summary, DTI is not yet ready for clinical use and requires extensive research to achieve this goal.
